# The Dual Role of Preexisting Immunity in Influenza: Protective Recall Versus Constraint of Novel Responses

**DOI:** 10.1111/imr.70146

**Published:** 2026-07-24

**Authors:** Annette Fox, Ziheng Zhu, Stephany Sánchez‐Ovando

**Affiliations:** ^1^ WHO Collaborating Centre for Reference and Research on Influenza, The Peter Doherty Institute for Infection and Immunity Royal Melbourne Hospital Melbourne Victoria Australia; ^2^ Department of Infectious Diseases, The Peter Doherty Institute for Infection and Immunity University of Melbourne Melbourne Victoria Australia

## Abstract

Preexisting immunity to influenza confers rapid protection against antigenically matched viruses but can also constrain responses to drifted variants. This review asks when and why prior infection or vaccination is protective versus detrimental, and which biological mechanisms—epitope masking by circulating antibody, inhibitory FcγRIIb signaling, and competitive dominance by affinity‐matured memory B cells—drive these outcomes. We synthesize human cohort, serological, and vaccine‐effectiveness data with mechanistic mouse and fate‐mapping studies to explore how vaccine formulation, antigen dose, antigenic distance and T cell help modulate the balance between memory recall and recruitment of naïve B cells. Finally, we outline strategies (higher dose, adjuvants, multivalent display, and considered strain selection) to restore de novo responses against escape epitopes and improve vaccine performance.

## 1 Current Seasonal Influenza Vaccines: Targets, Formulations, and Effectiveness

Neutralizing antibodies are key mediators of protection for most vaccines. The most potently neutralizing antibodies against influenza target epitopes surrounding the receptor binding site (RBS) in the exposed head of hemagglutinin (HA) and block virus attachment to sialic acids on host cells [1, 2, 3]. The hemagglutination inhibition (HI) assay detects antibodies that block virus attachment to sialic acids on red blood cells and has been established as a correlate of protection [4, 5]. Epitopes surrounding the RBS evolve rapidly by point mutation under positive selection pressure from antibody; this process, termed antigenic drift, can give rise to variants that infect previously exposed individuals.

The World Health Organization (WHO) coordinates global surveillance of influenza virus evolution and recommends vaccine strain composition updates annually based on antigenic and genetic data, including two‐way HI assays with antisera against vaccine and circulating strains. Vaccines are formulated to contain at least 15 μg of HA per strain. The most widely used vaccines comprise egg‐ or cell‐grown viruses that are inactivated and detergent split. Enhanced formulations include MF59‐adjuvanted and high‐dose (60 μg HA/strain) inactivated vaccines for older adults, recombinant HA protein vaccines (45 μg HA/strain), and live attenuated influenza vaccines administered intranasally. Meta analyses estimate overall vaccine effectiveness (VE) at around 60%, with substantially lower VE against A(H3N2) than A(H1N1) [6].

## 2 Development of Antibody Responses and the Impact of Antigen Formulation

Antibody titres rise after vaccination because antigen‐reactive B cells differentiate into antibody secreting plasmablasts and long‐lived plasma cells. During primary exposure, naïve B cells bind and internalize antigen via surface immunoglobulin (B cell receptor; BCR), then present peptides on MHC‐II to CD4^+^ T cells. The quality and amount of T cell help determine whether B cells enter germinal centers (GCs) or differentiate extrafollicularly into low‐affinity IgM plasmablasts or IgM memory B cells. GCs comprise a dark zone where B cells proliferate and undergo BCR somatic hypermutation, and a light zone where B cells bearing mutated BCR with higher affinity receive more T cell help and are selected for survival [7]. As B cells cycle through these zones and gain affinity they differentiate into memory B cells then plasma cells [7].

Affinity maturation, together with epigenetic changes that lower the costimulatory thresholds of memory B cells [8, 9, 10], markedly increases their propensity to differentiate into plasmablasts [10], facilitating faster and larger antibody responses during secondary exposure. This also explains why plasmablasts elicited by a variant vaccine strain are predominantly memory‐derived [11].

Studies in infants [12, 13, 14] and other species [15, 16, 17] show that humoral responses induced by primary exposure are strongest for infection followed by adjuvated‐ then unadjuvanted‐inactivated vaccine. This likely reflects stronger CD4^+^ T cell and GC responses following priming with live virus and adjuvanted vaccines compared to unadjuvanted inactivated vaccines [18, 19, 20].

## 3 Prior Infection Biases and Boosts Antibody Responses

Prior influenza infection enhances HI antibody responses to initial immunizations in children [21], and analogous findings occur in ferrets [17, 22], and mice [23]. These observations are consistent with weaker priming of immunity by inactivated vaccines than by infection. However, early life exposures may also bias or constrain responses to antigenically drifted strains encountered later in life [24, 25].

### 3.1 Evidence of Antibody Bias Towards Previously Encountered Strains

Francis and Davenport conducted seminal studies in the 1940s and 50's showing that antibody titres are typically highest against influenza strains encountered earliest in life [26, 27] and this persists following immunization with more recent strains [28]. These observations led to the original antigenic sin hypothesis that: “Upon subsequent exposure to influenza viruses of varied but related antigenic composition reinforcement of the level of antibody to the strains of primary infection occurs while the serologic response to prevailing viruses may be dampened” [29].

Contemporary cohort [30] and cross‐sectional studies [31] confirm and extend these findings by measuring HI antibody titres against A(H3N2) viruses spanning 1968 to at least 2008. Lessler et al. [31] proposed “antigenic seniority”, suggesting that memory B cells induced throughout life collectively dominate recall responses. Fonville et al. demonstrated that A(H3N2) infections back‐boost antibodies against previously recognized strains. The magnitude of back‐boost falls as antigenic distance from the infecting virus increases, consistent with reactivation of memory B cells when cognate epitopes are sufficiently conserved to engage B cell receptors. Consequently, antibody titres may be highest against viruses encountered early in life because each infection incrementally back‐boosts responses against antigenically related earlier strains. However, similar analyses of sera from two Australian vaccine studies suggest that this pattern of diminishing antibody titres against successively encountered strains could also reflect diminished de novo responses in the presence of preexisting antibodies [30]. In contrast, comparison of first‐time vaccine responses among adults with and without recent prior infection showed that postvaccination titres and titre rises were higher in those with prior infection despite having higher pre‐vaccination titres [32]. This highlights the complexity of prior immune effects where existing antibodies may signify the presence of memory B cells and be associated with enhanced responses or could inhibit B cell responses.

## 4 Antibody Responses and Clinical Impact of Repeated Vaccination

While inactivated vaccines induce robust antibody responses in previously primed children, whether repeated annual vaccination sustains antibody production and clinical protection is debated.

### 4.1 Vaccine Effectiveness (VE) Studies

Concerns about repeated vaccination emerged in the 1970s, when Hoskins found that inactivated whole‐virus vaccine protected boarding‐school boys who were vaccinated for the first time but not those who had been immunized previously with an earlier vaccine strain [33]. A 5‐year randomized trial in the 1980s found no consistent decline in whole‐virus vaccine efficacy with increasing prior vaccinations, although an increase in A(H3N2) infections was observed in one season [34]. The issue resurfaced around 2010 when a household cohort study reported lower VE among those vaccinated in consecutive seasons compared with single‐season vaccination [35, 36, 37]. Subsequent investigations revealed variable effects of prior vaccination suggesting that effects are modulated by subtype dominance and antigenic differences between vaccine strains and circulating strains [38, 39]. Meta‐analyses (covering 17 studies, 2009–2016 [39]; 41 studies, 2016–2022 [40]) show modest but significant reductions in VE associated with prior season vaccination, particularly for A(H3N2), albeit with substantial heterogeneity between seasons.

Few studies examine multiple prior vaccinations. Those assessing severe or hospitalized influenza showed no detrimental effect of vaccination during three to five prior seasons [41, 42]. Conversely, laboratory‐confirmed influenza studies consistently report reduced VE with vaccination during more than two prior seasons, predominantly for A(H3N2) [43, 44, 45, 46, 47].

### 4.2 Serological Studies

Serological studies reflect the heterogeneity seen in VE studies. Studies examining a single prior year of vaccination variably report attenuation, boosting, or no effect on antibody titres [48, 49, 50]. In contrast, studies of multiple consecutive annual vaccinations consistently report reduced postvaccination titres among participants receiving inactivated influenza vaccine for their fourth successive year or more compared with first‐ or second‐time vaccinees [51, 52, 53, 54, 55, 56]. In our healthcare worker cohorts, seroconversion rates have been as low as 5%–10% in individuals receiving their fourth consecutive vaccine, while remaining above 60% among first‐time vaccinees [54, 55].

Repeated vaccination is associated with attenuated HI antibody titres against both A(H1N1) and A(H3N2) [52, 53, 54, 55] whereas clinical impact is greatest for A(H3N2) [40]. A(H3N2) viruses exhibit greater antigenic heterogeneity and faster antigenic drift than A(H1N1) viruses [57] and postvaccination titres against vaccine strains likely overestimate protection since they are higher than against circulating A(H3N2) viruses, particularly among individuals who develop breakthrough A(H3N2) infections [32, 54, 58].

Taken together, the literature indicates that prior immune responses cause greater attenuation in the context of repeated vaccination compared to successive infections, possibly reflecting one or more of the following: lower immunogenicity of split inactivated vaccine formulations, shorter intervals between exposures with vaccination, or smaller antigenic distances between successive vaccine strains.

## 5 Preexisting Antibodies Limit B Cell Responses Against Cognate Epitopes but May Not Prevent Responses Against Escape Epitopes

Antibodies have an important role in delivering antigen to dendritic cells in order to induce T and B cell responses [59, 60], but it is also well established that antibody responses to inactivated vaccines decrease with increasing preexisting antibody titres [50, 53, 55, 61, 62, 63, 64, 65, 66]. Several of these studies show a concomitant decline in antigen‐specific plasmablast responses with increasing pre‐vaccination antibody titres [63, 64, 65].

**FIGURE 1:**
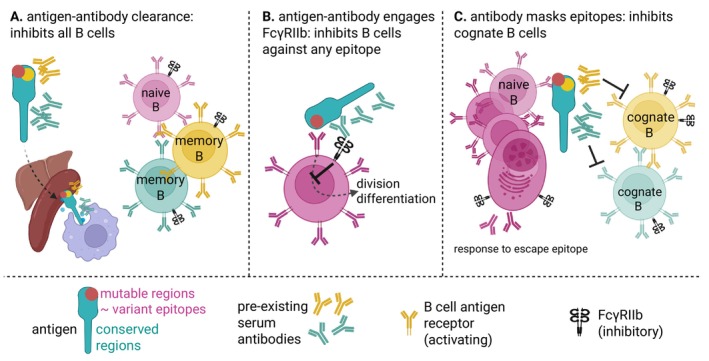
Potential mechanisms mediating antibody inhibition of B cells' responses. Created in BioRender. Fox, A. (2026) https://BioRender.com/njx1jd7.

A key question is whether preexisting antibodies suppress B cells broadly by clearing antigen (Figure 1A) or engaging inhibitory FcγRIIb receptors (Figure 1B) [67, 68] or more narrowly by masking cognate and neighboring epitopes [68, 69] but not necessarily escape epitopes (Figure 1C). A mathematical model based on human serology supports that antibodies against the head versus stalk regions of HA limit boosting against the matched regions only, consistent with epitope masking [68].

### 5.1 Passively Transferred Antibody Inhibits Primary B Cell Activation by Epitope Masking

Passive transfer of monoclonal antibody (mAb) to antigenic site Sb of A(H1N1) HA into naïve mice before immunization with inactivated virus suppresses GC responses, plasmablast formation and antibody production [2, 70]. Fab fragments limit suppression to Sb‐specific responses [2], whereas full‐length mAb additionally suppresses B cell responses to adjacent HA epitopes but not to neuraminidase [70]. Ablation of FcγR binding by full‐length mAb does not restore responses, indicating that antibodies block naïve B cell activation by masking cognate and adjacent epitopes [70].

### 5.2 Genetic Fate Mapping of Mouse Memory and De Novo B Cells During Recall Responses Shows Preexisting Antibodies Block Cognate but Not Escape‐Epitope Responses

**FIGURE 2:**
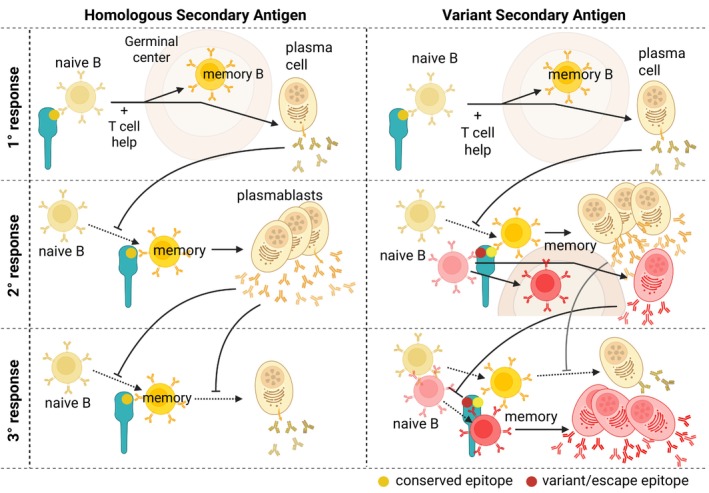
Effects of preexisting antibodies deduced from mouse models. Antibodies block germinal center B cell responses against cognate but not escape epitopes. Memory B cell differentiation into plasmablasts is refractory to low antibody titres but not to higher titres during tertiary responses. Created in BioRender. Fox, A. (2026) https://BioRender.com/xg1ul36.

It is more challenging to dissect whether preexisting antibodies inhibit memory and/or naïve B cell activation during recall responses. To address this, Victora and colleagues genetically engineered mice so that memory and de novo B cells, and the antibodies they produce, carry distinct Strep and FLAG tags. They show that secondary GCs are composed predominantly of naïve B cells and only a very small subset of the memory clones induced during priming [71]. However, there is limited activation of naïve GC B cells when priming and boosting employ identical HA proteins; by contrast, when the boosting antigen differs, de novo memory B cells are generated and contribute substantially to tertiary antibody responses [72]. By crossing fate‐mapped mice with mice that are unable to generate antibody secreting cells, they showed that de novo memory can be generated during secondary responses in the absence of preexisting antibody [73]. FcγRIIb signaling is not required for antibodies to suppress B cell responses in these models [73]. Hapten‐carrier experiments showed that help from memory T cells enables GC formation during secondary responses but does not overcome antibody‐mediated suppression of affinity maturation required to generate de novo memory [73]. These data support the inference that de novo memory can be induced against escape epitopes that are not masked by antibody. Victora et al. therefore propose that antibody‐mediated masking and suppression of cognate B cells directs de novo GCs towards variant‐specific epitopes (Figure 2). Fate mapping experiments also confirm that plasmablasts, and consequently antibodies, are mostly memory‐derived [71, 72]. Plasmablast production decreases during tertiary compared to secondary exposure [72, 74], and adoptive memory B cell transfer indicates that memory B cell differentiation is inhibited by high preexisting antibody titres (Figure 2) [74]. Hapten‐carrier experiments indicate that memory T cells alleviate antibody‐mediated inhibition of memory B cell differentiation [73]. This mechanism may in part explain why repeated vaccination is associated with particularly attenuated antibody responses since inactivated vaccines elicit relatively weak T cell responses [18, 19].

## 6 Evidence for Competing Mechanisms in Humans: Masking Versus Memory Dominance Versus Inhibitory Fc Engagement

Mouse models clearly show that preexisting antibodies can block cognate epitopes and thereby drive GC B cell responses towards escape epitopes. However, human data present a more complex picture. When only a single strain in a trivalent vaccine is updated, plasmablast responses in individuals vaccinated in consecutive years preferentially target the newly introduced variant [64]. Conversely, B‐cell receptor sequencing suggests that B cells responding to vaccination are recalled memory B cell clones rather than de novo clones that could target escape epitopes [75]. Additionally, frequent vaccination is associated with poor antibody responses across updated vaccine components [51, 52, 53, 54, 55], and with diminished detection of HA‐reactive B cells in GCs of draining lymph nodes [76].

Humans have complex exposure histories and possess antibodies against a relatively broad range of HA epitopes and strains [30, 77], which may restrict available escape targets and attenuate de novo responses relative to fate‐mapped mice. Other features of the mouse fate‐mapping experiments that may favor de novo B cell responses include high antigen doses (20 μg per mouse), use of adjuvant, recombinant HA protein rather than inactivated split virus, and two homologous boosts.

Human studies implicate factors beyond preexisting antibodies in the attenuation of antibody responses upon repeated vaccination: many repeatedly vaccinated individuals have low preexisting antibody titres, and repeat‐vaccination effects persist after adjusting or matching for baseline titre [50, 78, 79].

**FIGURE 3:**
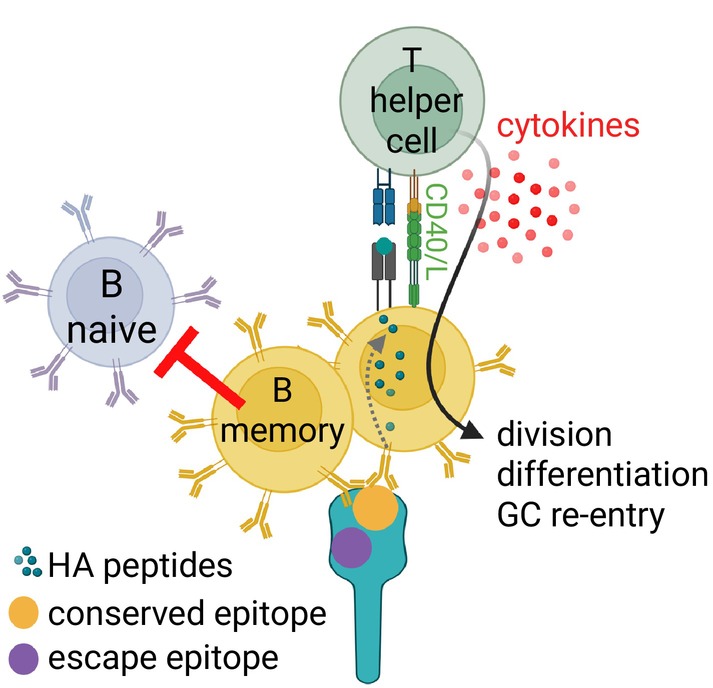
Competitive memory dominance hypothesis. Affinity matured memory B cells outcompete naive B cells for antigen and T cell help, which limits the generation of de novo responses against escape epitopes and focuses responses on conserved epitopes. Created in BioRender. Fox, A. (2026) https://BioRender.com/pbbusft.

Competitive memory B cell dominance (Figure 3) has been proposed as a mechanism that limits de novo B cell responses against escape epitopes. Affinity‐matured memory B cells are more effective at capturing antigen than naïve B cells and are already capable of co‐stimulating CD4^+^ T cells [8, 9]; consequently, memory B cells may outcompete naïve B cells for the T cell help needed to enter GCs [80]. This proposal followed reports that human antibody responses were focused on a rare epitope that was conserved between successively encountered A(H1N1) and A(H1N1)pdm09 strains [81, 82], and that HA‐reactive plasmablast responses in a few characterized participants were dominated by relatively few clones [74]. However, plasmablasts and their antibodies may not represent the full epitope breadth of responding B cells because they are largely derived from high‐affinity class‐switched memory B cells [18].

**FIGURE 4:**
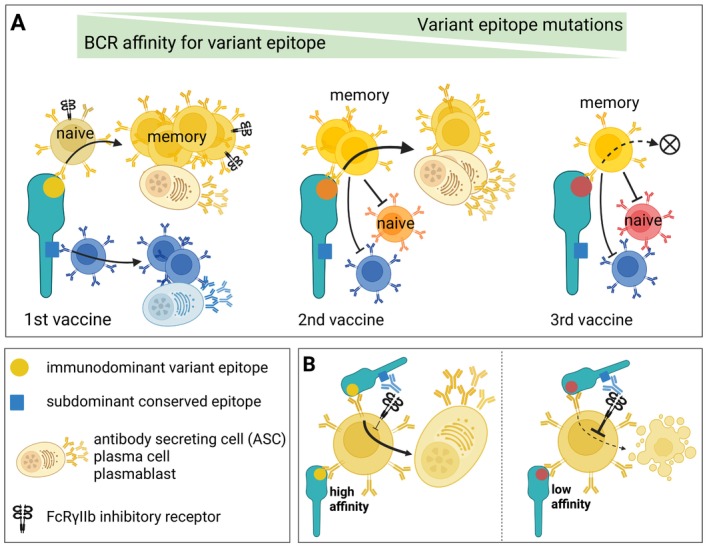
Proposed mechanisms for loss of memory B cell responsiveness after successive vaccinations. (A) memory B cells against immunodominant HA head epitopes monopolize antigen and T cell help; as these epitopes mutate, memory BCR affinity and response magnitude decline but may still prevent de novo memory formation. (B) high affinity BCR engagement leads to memory B cell activation, whereas low affinity engagement of mutated epitopes combined with inhibitory FcγRIIb engagement by preexisting antibodies may drive memory B cell inhibition or death. Created in BioRender. Fox, A. (2026) https://BioRender.com/95jywz1.

In contrast to the memory dominance hypothesis, our recent (unpublished) studies indicate diminished activation, expansion, and differentiation of HA‐reactive memory B cells among repeatedly vaccinated healthcare workers. We assessed 876 samples from 147 healthcare workers by flow cytometry using recombinant HA antigens. Of these participants, 62 had not received influenza vaccination during the 5 years prior to enrolment, whereas 95 had been vaccinated every year. One possibility is that memory B cells continue to bind their cognate epitopes in updated vaccine strains and thereby limit de novo responses, but with epitope mutation their BCR affinity decreases and their responsiveness declines (Figure 4A). In addition, low‐affinity engagement of the BCR by antigen that is complexed with preexisting antibody may further suppress memory B‐cell responses because engagement of FcγRIIb in the absence of productive BCR co‐crosslinking can deliver inhibitory signals and promote apoptosis (Figure 4B). Consistent with this model, FcγRIIb expression is higher on memory than on naïve B cells [9, 83, 84] and antibody responses are augmented in FcγRIIb‐deficient mice [59].

## 7 Strategies to Improve Immunity Towards Updated Vaccine Strains

The combined evidence indicates that inactivated influenza vaccines efficiently drive memory B cell differentiation, producing plasmablasts and antibodies, but they weakly activate naïve B cells. Existing memory B cells may further restrict naïve B cell activation and the formation of de novo memory against escape epitopes. While existing antibodies could drive de novo GC B cell responses towards escape epitopes, this may require larger antigen doses approximating those used in fate mapped mouse experiments or be contingent on existing antibody diversity and the extent of antigenic change between vaccine strains. Thus, strategies are required that increase the intrinsic capacity of vaccines to recruit naïve B cells while preserving memory B cell activation and antibody production.

It is clear that the immunogenicity and efficacy/effectiveness of inactivated vaccines is improved by increasing antigen dose or by adding adjuvants [85], which increase the effective antigen dose [85]. Whether enhanced vaccines augment de novo responses and restore vaccine immunogenicity in the context of repeated vaccination is less certain. A randomized trial in healthy adults reported attenuated humoral responses among repeatedly vaccinated individuals who received standard‐dose egg‐ and cell‐grown vaccines, but not among those given a recombinant HA vaccine containing three times more HA [79]. In older adults, however, recombinant, adjuvanted and high‐dose vaccines all improved immunogenicity, yet the attenuating effect of repeated vaccination persisted [50, 78].

Mechanistically, higher antigen doses may overwhelm preexisting memory B cells and antibodies and lower the minimal B cell‐receptor affinity threshold for GC selection, thereby favoring naïve B cell entry [86]. Similarly, multivalent assemblies of subunit proteins such as viruslike particles cross‐link BCR and reduce the minimal BCR affinity required for activation [87]. Larger antigens such as virus‐like particles are also more readily transferred to follicular dendritic cells, increasing the effective antigen dose [88]. Naïve B cell activation also requires T cell help and engagement of innate inflammatory pathways [10]. Together, these data suggest that vaccines that combine high antigen doses with adjuvants that target costimulatory pathways of naïve B cells may be required to generate de novo responses in the context of repeated vaccination.

In addition, vaccine strain selection strategies could be employed to reduce attenuation from repeated vaccination. Support for increasing antigenic distance between vaccine strains comes from mathematical models, animal studies and limited vaccine study data [30, 38]. There was a dramatic increase in VE against A(H1N1) in the 2020/21 season, coinciding with substantial antigenic drift from prior vaccine strains [89]. We assessed HI antibody responses of 120 highly vaccinated healthcare workers during 2020 through 2022 and observed a corresponding recovery of antibody and B cell responses in the 2021 season (unpublished). Trials that compare the immunogenicity of conservative versus antigenically advanced vaccine strains are needed to determine whether this strategy could induce de novo responses while maintaining memory B cell boosting and antibody responses.

## 8 Concluding Remarks

Taken together, the available human and experimental data indicate that preexisting influenza immunity can confer a rapid, protective effect. The outcome is determined by antigenic distance, antigen dose, vaccine formulation, exposure history, and the quality of T cell help. By contrast, under conditions of repeated exposure to split inactivated vaccines with relatively low antigen doses, limited antigenic change, and weak T cell help, high baseline antibodies and memory B cells may attenuate responses through the mechanisms outlined in this review.

These insights suggest that optimizing vaccine performance in highly exposed populations will require strategies that use higher antigen doses, multivalent displays, and adjuvants that enhance naïve B cell GC entry, as well as strain selection approaches that favor antigenically distant vaccine strains to restore de novo responses while preserving the benefits of memory recall.

## Conflicts of Interest

A.F. reports funding/grants from Sanofi Aventis, France. Otherauthors declare that there are no conflicts of interest regarding the publication of this paper.

## Data Availability

Data sharing not applicable to this article as no datasets were generated or analyzed during the current study.
